# Integrative Study of the Structural and Dynamical Properties of a KirBac3.1 Mutant: Functional Implication of a Highly Conserved Tryptophan in the Transmembrane Domain

**DOI:** 10.3390/ijms23010335

**Published:** 2021-12-29

**Authors:** Charline Fagnen, Ludovic Bannwarth, Iman Oubella, Dania Zuniga, Ahmed Haouz, Eric Forest, Rosa Scala, Saïd Bendahhou, Rita De Zorzi, David Perahia, Catherine Vénien-Bryan

**Affiliations:** 1 IMPMC, UMR 7590, CNRS, Muséum National d’Histoire Naturelle, Sorbonne Université, 75005 Paris, France; charline.fagnen@unicaen.fr (C.F.); l_bannwarth@hotmail.com (L.B.); i.oubella@hotmail.fr (I.O.); dania.zuniga@sorbonne.universite.fr (D.Z.); 2 Laboratoire de Biologie et Pharmacologie Appliquée, Ecole Normale Supérieure Paris-Saclay, 4 Ave. des Sciences, 91190 Gif-sur-Yvette, France; david.perahia@ens-paris-saclay.fr; 3 Institut Pasteur, C2RT-Plate-Forme de Cristallographie CNRS-UMR3528, 75724 Paris, France; ahmed.haouz@pasteur.fr; 4 CNRS, IBS, CEA, University Grenoble Alpes, 38044 Grenoble, France; eric.forest@ibs.fr; 5 CNRS UMR7370, LP2M, Labex ICST, Faculté de Médecine, University Côte d’Azur, 06560 Nice, France; rosa.scala@univ-cotedazur.fr (R.S.); said.bendahhou@univ-cotedazur.fr (S.B.); 6 Department of Chemical and Pharmaceutical Sciences, University of Trieste, Via Licio Giorgeri 1, 34127 Trieste, Italy; rdezorzi@units.it

**Keywords:** crystal structure of KirBac3.1 W46R, HDX-mass spectrometry, molecular dynamics, normal modes, electrophysiology, gain of function Kir, neonatal diabetes mellitus

## Abstract

ATP-sensitive potassium (K-ATP) channels are ubiquitously expressed on the plasma membrane of cells in several organs, including the heart, pancreas, and brain, and they govern a wide range of physiological processes. In pancreatic β-cells, K-ATP channels composed of Kir6.2 and SUR1 play a key role in coupling blood glucose and insulin secretion. A tryptophan residue located at the cytosolic end of the transmembrane helix is highly conserved in eukaryote and prokaryote Kir channels. Any mutation on this amino acid causes a gain of function and neonatal diabetes mellitus. In this study, we have investigated the effect of mutation on this highly conserved residue on a KirBac channel (prokaryotic homolog of mammalian Kir6.2). We provide the crystal structure of the mutant KirBac3.1 W46R (equivalent to W68R in Kir6.2) and its conformational flexibility properties using HDX-MS. In addition, the detailed dynamical view of the mutant during the gating was investigated using the in silico method. Finally, functional assays have been performed. A comparison of important structural determinants for the gating mechanism between the wild type KirBac and the mutant W46R suggests interesting structural and dynamical clues and a mechanism of action of the mutation that leads to the gain of function.

## 1. Introduction

The inwardly rectifying potassium channels (Kir channels) regulate membrane electrical excitability and K^+^ transport in a wide range of cell types. They control various processes, including vascular tone, heart rate, and insulin secretion. Dysfunctional Kir channels are directly associated with a growing number of diseases [1]. Kir channel activity is controlled by dynamic conformational changes that regulate the flow of K^+^ ions through the central pore of the channel [1,2]. This process is known as “gating”, and understanding the molecular mechanism behind these dynamic changes in the structure of Kir channels is essential to understanding how these channels function for both health and disease.

Kir6.1 and Kir6.2 are typical Kir channels; they have two transmembrane helices, TM1 and TM2, linked by a pore loop, and intracellular amino and carboxy termini. When associated with sulfonylurea receptor proteins (SUR1 or SUR2, belonging to the ABC (ATP-binding cassette) transporter superfamily), they form the ATP-sensitive potassium (K-ATP) channels. K-ATP channels are primarily expressed in the pancreatic endocrine islets, brain, cardiac myocytes, skeletal muscle, and vascular smooth muscle [3]. K-ATP channels consist of the pore-forming subunits Kir6.1 and Kir6.2, and auxiliary subunits SUR1, SUR2A, and SUR2B. Four SURs and four Kir6.x subunits form an octameric channel complex. The association of a specific SUR with a particular Kir6.x subunit forms the K-ATP of a particular tissue. They have various physiological roles, including controlling the release of insulin from pancreatic β cells and regulating blood vessel tone and blood pressure.

K-ATP channels are so called because they open when cellular ATP levels fall, thus linking cellular metabolism to membrane excitability. They also respond to Mg-ADP and are regulated by several cell signaling pathways [1,4,5]. K-ATP channels detect metabolic changes via three classes of ATP/ADP binding sites. One site, located on the Kir subunit, causes inhibition of the channel (closes the channel) when ATP is bound. The other two sites, located on the NBD (nucleotide-binding domain) of SUR subunits, bind to Mg^2+^-ATP and Mg^2+^-ADP, causing dimerization of the NBDs, which counteracts the inhibitory action of ATP on Kir6.2 and thereby stimulates the channel [6].

Blood glucose levels determine ATP and ADP concentrations. When glucose levels rise, the intracellular ATP-to-ADP ratio increases due to glycolysis, and ATP inhibition dominates suppressing K-ATP channel activity. In contrast, the ATP-to-ADP ratio decreases when blood glucose levels drop and MgADP stimulation prevails, increasing channel activity. Loss-of-function mutations cause hyperinsulinemic hypoglycemia of infancy, characterized by persistent and unregulated insulin secretion and life-threatening hypoglycemia. Conversely, gain-of-function mutations alter insulin secretion and produce neonatal diabetes mellitus, which manifests within the first six months of life. Because the K-ATP channel is also expressed in the brain, patients with functionally severe mutations can exhibit neurological symptoms in addition to neonatal diabetes mellitus, a condition known as DEND syndrome [7,8,9,10,11,12].

KirBac3.1, Kir6.2, and Kir6.1 channels are homotetramers of four subunits. Each subunit has a transmembrane domain (TMD), containing an outer transmembrane helix (TM1), a selectivity filter (SF), and an inner transmembrane helix (TM2). Besides the TMD, there is a cytoplasmic domain (CTD) composed of N- and C- termini located on the intracellular side of the membrane.

A novel heterozygous-dominant mutation, W68R, in the Kir6.2 subunit of the K-ATP channel in a patient with transient neonatal diabetes, developmental delay, and epilepsy, has been identified [13]. This tryptophan residue at the cytosolic end of TM1 is very well conserved across the prokaryotic and eukaryotic, including mammalian Kir family and is found at the position W46 in KirBac3.1.

In this paper, we present the crystal structure of the KirBac3.1 W46R mutant channel (homologous to the human Kir6.2 channel) at 2.80 Å and provide a structural and functional characterization using a multi-technique approach. The mutation replaces a tryptophan residue that is highly conserved across the Kir family to an arginine, disrupts the contacts between the outer (TM1) and inner (TM2) helices, and creates new contacts shown in our crystallographic data. HDX-MS experiments show differences in the conformational flexibility of the W46R mutant when compared with the KirBac3.1 WT. Besides, *in silico* molecular dynamics studies allow us to investigate in detail the influence of this mutated residue on the molecular mechanism of gating of the channel. Moreover, this mutated channel’s open probability (Po) has been investigated through functional experiments, finally corroborating the *in silico* investigations.

## 2. Results

### 2.1. Crystal Structure of KirBac3.1 W46R

**Figure 1:**
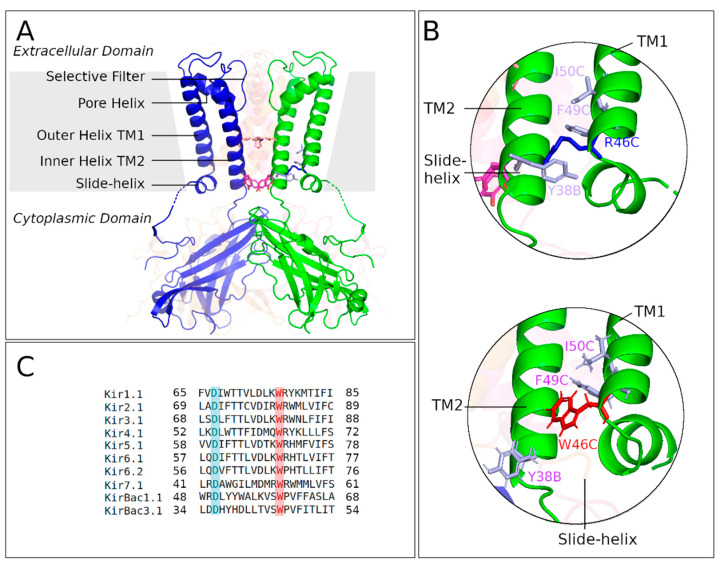
Crystallographic structure of KirBac3.1 W46R (PDB 7ADI). (**A**) Structure of KirBac3.1 W46R showing the location of the mutant R46 at the bottom of TM1. The gray area represents the membrane, and arrows indicate the various domains in the figure. Orange and magenta residues at the center of the channel correspond to the constriction points Leu124 and Tyr132, respectively. For clarity, two subunits out of four are represented. (**B**) Top: Zoom on the area around the mutation R46 (blue) at the bottom of TM1 from chain C (R46-C) interacting with Y38-B on the slide helix of the n-1 chain (chain A counterclockwise neighbor chain). Bottom: Same area as above with KirBac3.1 WT (PDB 2WLJ). W46-C (chain C, in red) in the “flipped-in” conformation packed against the C-terminal end of TM2 (chain B). (**C**) Alignment for Kir channels sequences. The tryptophan residue at the position corresponding to W68 in Kir6.2, at the bottom of TM2, is absolutely conserved (pink). The aspartate residue at the position corresponding to D58 in Kir6.2, on the slide helix is also highly conserved (cyan); note that the Y38 (KirBac3.1) is equivalent to F61 (Kir6.2).

The tryptophan at position 46 in KirBac3.1, corresponding to W68 in Kir6.2, located at the cytosolic end of transmembrane helix 1 (TM1) (Figure 1C), is highly conserved within the inward rectifying potassium channel family, suggesting that it has a critical role.

We expressed the mutant W46R of KirBac3.1, corresponding to pathological forms of the human homologous Kir6.2 channel, and investigated this mutant’s high-resolution structure. The R46 mutation replaces the hydrophobic tryptophan and introduces a positively charged residue. The KirBac3.1 W46R mutant channel was expressed, purified, and crystallized, and the structure was solved and refined to 2.80 Å resolution. We grew crystals of the W46R mutant belonging to space group P2_1_2_1_2. The structure was solved by molecular replacement, and a final model containing 279 out of the 301 residues of the construct was refined to an R-factor of 22.2% and an R-free of 28.7%, with 99.3% of the residues in the final model lying either in the most favorable or in the allowed regions of the Ramachandran plot. The complete data collection and refinement statistics are shown in Table 1, and the crystallographic structure is shown in Figure 1A. A backbone alignment with the structure of the KirBac3.1 WT (PDB 2WLJ) [14] showed that the two structures were very similar (root mean square deviation, rmsd = 0.471 Å). Both structures, WT and W46R, were in a closed state.

Interestingly, despite this overall similarity, significant differences were evident in the vicinity of residue 46 (W or R) (Figure 1B). As a consequence of the mutation, a series of changes occurred in the inter- and intrasubunit interactions at the level of the bundle crossing at the bottom of the transmembrane helix compared with the KirBac3.1 WT (Figure 1B). In most X-ray structures of WT Kir channels, the residue corresponding to W68 in Kir6.2 is in the “flipped-in” conformation tightly packed against the C-terminal end of TM2, as seen in the crystal structure (Figure 1B bottom, residue marked as W46 according to KirBac3.1 numbering). As a result, the W46 side chain interacts with the F135 and R134 at the bottom of the TM2 (See Section 2.3.1). More seldom, the tryptophan side chain can adopt a different rotameric conformation (“flipped-out”), pointing away from the channel pore and removing the interaction with the bottom end of TM2 (Kir2.2, PDB 3JYC). The atomic structure of the W46R mutant shows that the four R46 side chains point away from the channel pore and are engaged in intersubunit connections with residues Y38 and D36 on the slide helix of the adjacent subunit (n − 1: counterclockwise neighbor chain) (Figure 1B top).

### 2.2. HDX-MS Measurements

**Figure 2:**
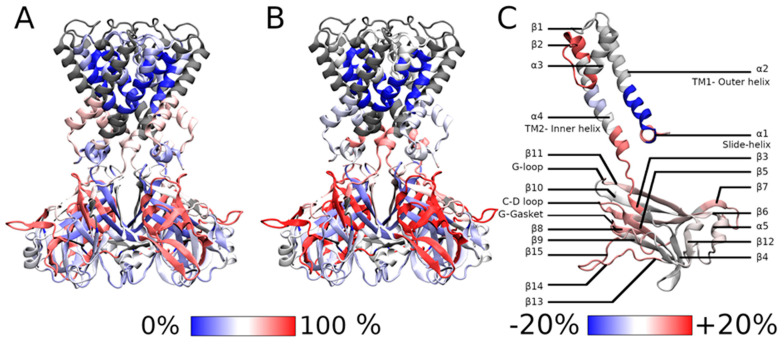
HDX-MS rates. (**A**) HDX-MS rates of peptides that we have reported for the KirBac3.1 WT [18] and (**B**) for the mutant W46R. (**C**) Rate difference between the W46R mutant and KirBac3.1 WT. The scale of the deuterium exchange rate is shown at the bottom of the figures, with blue corresponding to low exchange rate and high rigidity; and red to high exchange rate and high flexibility.

The conformational flexibility of KirBac3.1 W46R mutant was investigated using HDX-MS. This technique is based on the exchange of deuterium atoms at the amide backbone of a protein, reflecting its conformational dynamics, followed by proteolytic digestion and spectrometry analysis [15,16,17]. HDX has already been extensively used on soluble and membrane proteins [18,19,20]. HDX was performed on KirBac3.1 W46R mutant in the presence of detergent and compared to KirBac3.1 WT also in detergent and recently reported [18], Figure 2A. In previously published work, we have established that the presence of detergent does not affect the conformational changes of the KirBac3.1 channel [21]. We tested digestion with various proteases to achieve sequence coverage as extensively as possible. The optimized conditions resulted in 86% sequence coverage with nepenthesin. However, this enzyme did not allow coverage of regions 57–87 (second half of the TM1 and the beginning of the pore helix), 143–147 (β3), and 195–199 (second half of the β7). Deuterium incorporation was monitored as a function of time for each peptide generated from the W46R mutant (Figure S1).

Our data show that the most flexible regions for KirBac3.1 W46R are the loops extending outside the CTD (aa 271-285 between β14 et β15, in red in Figure 2B, see Figure 2C for the nomenclature) with an HDX rate of 68.0%. The next, more flexible, three domains are: (i) at the G-gasket just below the CD-loop and the entire β8, (aa 199–210, with HDX rate of 66.0%); (ii) the stretch going from the end of the β5 and the start of the β6 including the CD-loop in the middle (aa 162–174, HDX rate of 65.7%); and (iii) the stretch going from the end of β6 and the start of β7 including the facing-down loop in the middle (aa 181–194, with HDX rate of 64.1%). On the CTD, the stretch including the end of the G-loop and the following β11 also shows some flexibility (aa 250–260, HDX rate of 43.1%).

In the transmembrane domain, the stretch at the bottom of TM2-loop and β3, which includes the Y132 restriction point, shows some flexibility (aa 132–143, with an HDX rate of 53.7%). In contrast, the top of the TM2 is exceptionally stiff, while the slide helix also shows less flexibility (aa 36–42, with an HDX rate of 28.9%).

We compared the conformational dynamics of the W46R mutant with the KirBac3.1 WT (Figure 2C). In the figure, the blue color on the structure indicates a higher rigidity for the W46R mutant than the WT, while red stretches represent regions where the mutant shows increased conformational flexibility. Interestingly, the stretch that includes the very end of the slide helix, R46 mutation, and the bottom half of the TM1 external helix (aa 43–56) is not as flexible as the WT (normalized HDX rate of 37.9% compared with 65.1%). The interactions network around the R46 residue is remarkably stable. This stretch including R46 is also the only part of the mutant protein which is more rigid than the WT. Conversely, the stretch of aa 36–42 before the mutation and including most of the slide helix (except the very end) is more flexible than in the WT (normalized HDX rate of 41.57% compared with 26.92%). This difference is the largest of all the peptides analyzed.

Higher flexibility induced by the presence of the mutation has also been observed for other parts of the protein: (i) on the stretch including the bottom of the pore helix, the selectivity filter, and the top of the external helix (aa 93–112, normalized HDX rate of 50.04% for the mutant compared with 30.08% for the WT); (ii) on the stretch corresponding to the bottom of the internal helix and a large loop just before the β3 (aa 131–143, normalized HDX rate 77.32% compared with 61.26%) where the increased deuterium exchange rate could be interpreted as a higher accessibility surface; (iii) on the stretch corresponding to sheets β5 and β6 in the cytoplasmic domain and CD loop (aa 162–174, normalized HDX rate 94.61% compared with 83.1%); and (iv) on the segment that includes the G-loop which is slightly more flexible in the case of the mutant (aa 250–260, normalized HDX rate 62.05% compared with 55.25%).

### 2.3. Theoretical Results on KirBac3.1 W46R

The theoretical results presented in this section are based on MDeNM simulations, in which a selected set of normal modes (NMs) related to channel gating are excited in molecular dynamics (MD) simulations [22]. Through a combination of MD and NMs, this method is a realistic exploration of the relevant normal mode space for the gating processes taking into account the full environment (water, ions, and membrane) of the protein at a given temperature. These simulations were supplemented by standard MD simulations on a uniformly distributed set of structures obtained from MDeNM that provide a reasonably good estimation of the open/closed (and partially open) states populations as previously reported [18,23].

#### 2.3.1. Interaction Networks

**Figure 3:**
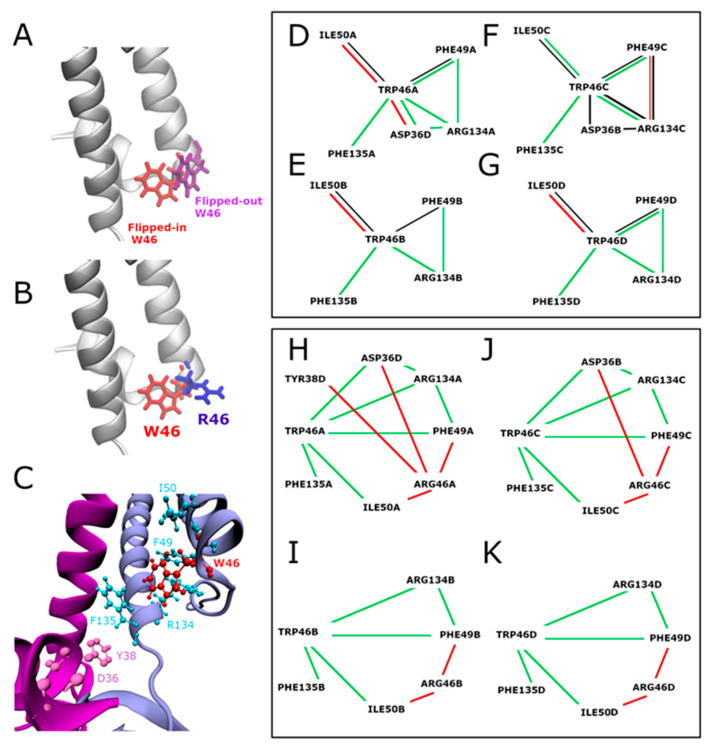
Overview of the network of interactions around W46 (KirBac3.1 WT) and R46 (KirBac3.1 W46R). (**A**) KirBac3.1 WT: representation of the W46 flipped-in (in red, from X-ray structure PDB 2WLJ) and the flipped-out (in purple, from a representative structure issued from MDeNM simulations [18]. (**B**) Superimposition of the residue W46 (flipped-in) in red and the mutation R46 in blue from our crystallographic structure. (**C**) Network of interactions around W46. The chains A and D are colored in blue and magenta, respectively, and the residue W46 (flipped-in) is in red. (**D**–**G**) KirBac3.1 WT: Comparison of the interaction network of residue W46 in flipped-in and flipped-out conformations for the chains (**A**) (panel (**D**)), (**B**) (panel (**E**)), (**C**) (panel (**F**)), (**D**) (panel (**G**)). The green lines indicate the interactions present in the flipped-in configuration but absent in the flipped-out one, and red lines those present in the flipped-out configuration but absent in the flipped-in one. Black lines are shared between the two conformers. (**H**–**K**) Comparison of the interaction network of residue W46 flipped-in (KirBac3.1 WT) and residue R46 (KirBac3.1 W46R) for the chains (**A**) (panel (**H**)), (**B**) (panel (**I**)), (**C**) (panel (**J**)), (**D**) (panel (**K**)). The green lines indicate the interactions present in KirBac3.1 WT but absent in the W46R mutant, and the red lines the interactions present in the W46R mutant but absent in KirBac3.1 WT. For more details, see Figure S2.

Interaction network analyses were performed on the crystallographic structures of KirBac3.1 WT (PDB 2WLJ) [14] and on the KirBac3.1 W46R mutant model (see Materials and Methods) to elucidate the impact of the mutation W46R on the structural and dynamical properties of the protein. The residue W46 in KirBac3.1 WT can adopt two alternative conformations, called flipped-in, in the majority of cases (side chain of W46 oriented towards the channel) and flipped-out (side chain of W46 oriented towards the outside of the channel) (Figure 3A). Figure 3C presents details on the flipped-in structure. The atomic structure of the W46R mutant shows that the four R46 side chains point away from the channel pore in the crystal structure of the mutant W46R (Figure 3B). This is also observed in the computational structures of KirBac3.1 W46R (98.68% of the 25415 relaxed MDeNM structures).

The first analysis focused on the KirBac3.1 WT: we compared the interaction networks (hydrogen bonds, van der Waals interaction, π-π and π-cation interactions) at the W46 residue level in flipped-in and flipped-out configurations. Interaction networks around W46 for these two configurations are shown in Figure 3D–G respectively for chains A, B, C, and D, separately (for more details, see Figure S2). W46 is tightly packed against the C-terminal end of TM2 of the same subunit and interacts with R134 and F135 at the bottom of this inner helix in the flipped-in conformation. Conversely, in the flipped-out conformation, these interactions are not possible, confirming the movement of the W46 residue away from the bottom of the inner helix and the center of the channel.

The second analysis focused on comparing the interaction network around the W46 residue, in its flipped-in conformation (the most frequent conformation) in KirBac3.1 WT and the interaction network around R46 in the W46R mutant (Figure 3H–K and Figure S2 for more details). Some similarities are evident, e.g., in all the four subunits, corresponding to chains A, B, C, and D, both W46 and the mutated R46 residues interact with the residues I50 and F49 on the same external helix TM1. However, there are some clear differences. The interactions between the flipped-in W46 residue and the two R134 and F135 residues at the bottom of the TM2 are lost in the case of the W46R due to the orientation of the mutated residue R46 toward the outside of the channel. Along with this loss of interactions, a new network around R46 is established and interestingly new interactions can take place with D36 and/or W38 residues in the slide helix of the adjacent subunit (n − 1). It is important to highlight that only R46 residues from two opposite chains (A and C) interact with the slide helix. Such contact is not evident for the other two opposite chains (B and D). These observations suggest that the mutation R46 alters the interaction of the outer and inner helices at the level of the bundle crossing, disrupts a hydrophobic cluster (Figure S3), and promotes the interaction of the bottom end of the outer external helix with the slide helix of the adjacent (n − 1) subunit. The R46 side chain points towards the outside of the channel and shows similarities with the flipped-out configuration of W46 residues in the wild type (WT).

#### 2.3.2. Contacts between Residues

- Accessible surface of the residues W46 and R46

**Figure 4:**
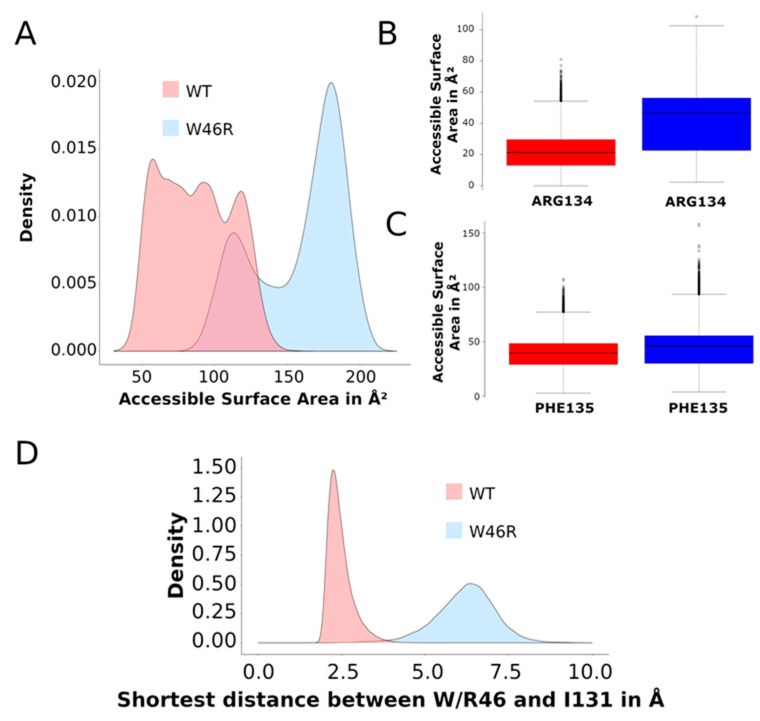
Accessible surface areas and shortest distances on MDeNM structures. (**A**) Histogram of accessible surface area (ASA) values of residue W46 in KirBac3.1 WT (in red) and R46 in KirBac3.1 W46R (in blue) obtained on the set of relaxed MDeNM structures. (**B**) Boxplot of ASA values of R134 in KirBac3.1 WT (in red) and in KirBac3.1 W46R (in blue) over the same set of structures. (**C**) Boxplot of ASA values of F135 in KirBac3.1 WT (in red) and in KirBac3.1 W46R (in blue) over the same set of structures. (**D**) Histogram of the shortest distances on MDeNM structures between residue 46 and I131 in KirBac3.1 WT (in red) and KirBac3.1 W46R (in blue).

We calculated the accessible surface areas (ASAs) of residue 46 (W in WT and R in the W46R mutant) on all the 25,415 relaxed MDeNM structures. This value measures the residue’s interactions in this position and its degree of exposure to the solvent. The histograms of the ASA values given in Figure 4A show that R46 in the mutant is more exposed than W46 in WT. The corresponding average value is 87.42 Å² with a standard deviation (SD) of 24.87 Å² for the WT and 158.7 Å² (SD = 30.81 Å²) for the mutant. These values are in accordance with our crystallographic data showing that the residue R tends to point out of the channel. We should also consider the different hydrophobicity of the two residues. R is more hydrophilic than W and therefore has more favor interactions with the water environment or the polar heads of the phospholipids.

- Accessible surface of the residues R134 and F135 and contacts with I131

In the closed flipped-in state, the side chain of W68 in Kir6.2 (PDB 6JB1) is directed towards the C-terminal end of TM2 and holds hydrophobic contacts with residues I167, K170, and T171 [24,25]. In the homologous KirBac3.1 channel, W46, corresponding to W68 in Kir6.2, is also directed to the C-terminal end of TM2 (Figure 1B and Figure 2C) and is close to the I131 (corresponding to I167 in Kir6.2), R134 (corresponding to K170 in Kir6.2), and F135 (corresponding to T171 in Kir6.2).

In KirBac3.1, the proximal residues R134 and F135 are more exposed to the solvent in the mutant W46R than in the WT (Figure 4B,C), supporting the suggested loss of interaction with R46 in the mutant protein. It is worth noting the significant increase in ASA value for R134 compared to F135. R134 shows an ASA average value of 21.77 Å² (SD = 10.96 Å²) in the KirBac3.1 WT and 41.05 Å² (SD = 18.19 Å²) in the mutant W46R. F135 shows an average value of 38.90 Å² (SD = 13.87 Å) and 44.27 Å² (SD = 17.59 Å²) in KirBac3.1 WT and W46R mutant, respectively. These results are explained by the fact that R134 is closer to the bottom end of TM2 and, therefore, more accessible to the solvent. It is also interesting to note that R46 has a higher ASA value than R134, indicating the significant exposure and flexibility of the latter.

The distribution of shortest distances between residues W46/R46 and I131 calculated on the MDeNM set of conformations shows that the shortest distance is higher (for all the chains) in the mutant (Figure 4D). The average values are 2.45 Å (SD = 0.37 Å) and 6.20 Å (SD = 0.9 Å) for the WT and the W46R mutant, respectively.

All the evidence gathered with this study points to a clear disruption of these crucial interactions between TM1 and TM2 in the case of the W46R mutant, compared to the WT protein.

#### 2.3.3. Impact of the Mutation on the Gating

In order to investigate the effects of the mutation W46R on various dynamical components of the gating mechanism from the selected normal modes described in Materials and Methods, we carried out MD simulations using MDeNM including the entire environment (water, lipids, ions). Structural and dynamical features taken into account are (i) the movement of the slide helix of each chain, (ii) the movement of the whole cytoplasmic domain, and (iii) the coordinated movement of the transmembrane helices.

- Upward movement of the slide helix

**Figure 5:**
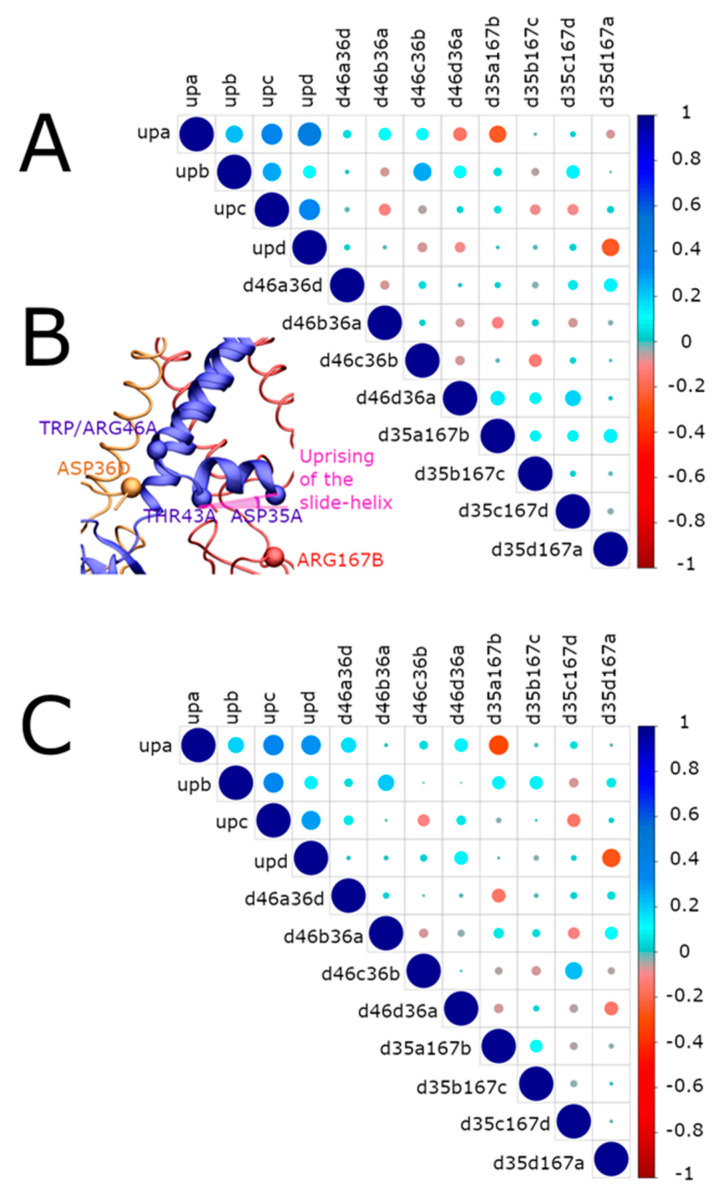
Correlation between the interactions of the slide helices and their upward movement in KirBac3.1 W46R and WT. (**A**) Correlations in the W46R mutant. The upward movement of the slide helix for the chains A, B, C, and D is denoted upx, where x is the letter of the chain. D46n36n-1 corresponds to the shortest distance between the R46 residue of the chain n and the D36 residue of the chain n − 1, and d35n167 n + 1 to that between the D35 residue of the chain n and the R167 residue of the chain n + 1. The blue circles correspond to positive correlations, while the red ones correspond to negative correlations. The diameter and the color intensity of a circle are proportional to the strength of the correlation. (**B**) Locations of the residues and the slide helix upward movement angle definition are considered in the correlations in pink. (**C**) Correlations in the case of KirBac3.1 WT.

The W46R mutation’s impact on the motion of the slide helix was analyzed considering the interaction of the R46 residue with residues of the slide helix of the adjacent protein monomer (n − 1). To this end, we calculated the correlations between the upward movement of the slide helices (definition of the angle in Material and Methods and Figure 5B, in pink) and the shortest distances between R46 at the extremity of TM2 and D36 located at the extremity of the slide helix. This movement has been previously highlighted as important in the gating of the channel [18]. Figure 5A shows the positive correlation between helix movements and inner-residues distances in blue circles, while red circles indicate negative correlations. The diameter and color intensity of each circle are proportional to the strength of the correlation. The shortest distances between R46 and D36 of neighboring chains (n − 1) are mainly correlated with the upward movement of slide helices. Therefore, it can be inferred that a smaller distance between these two residues induces a larger upward movement of slide helices.

**Figure 6:**
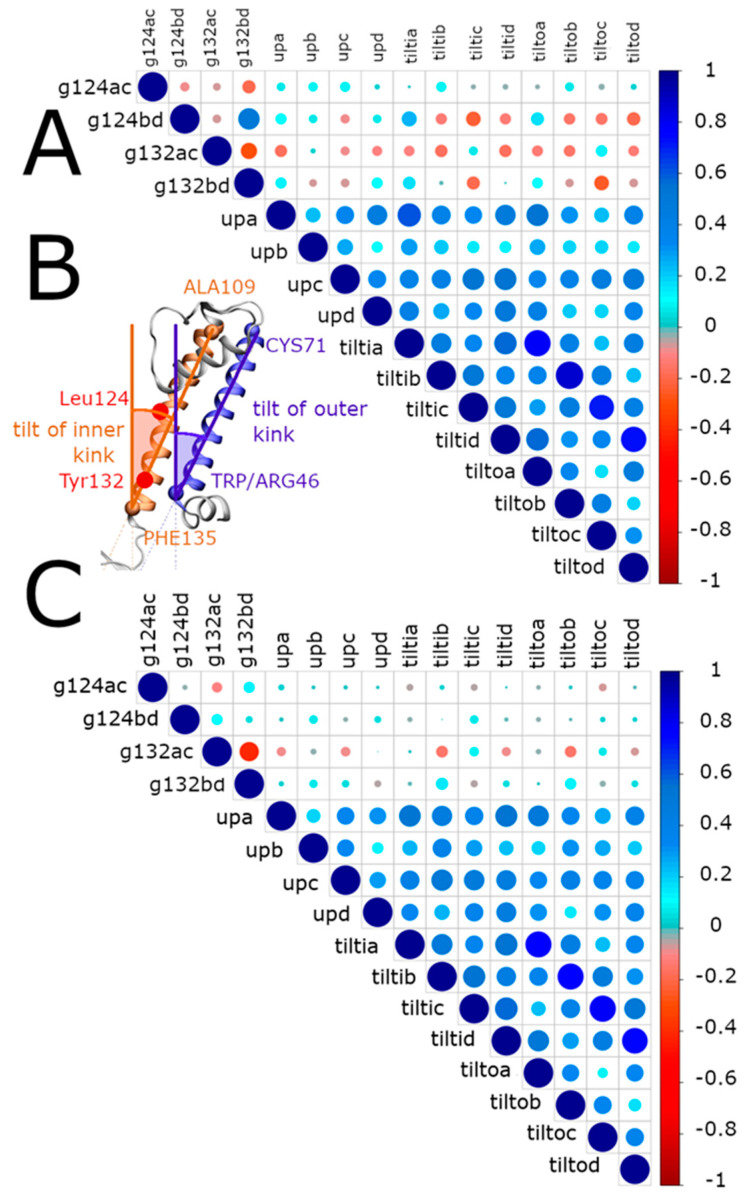
Correlation between the gating and the upward movement of the slide helices and inclinations of the transmembrane helices in KirBac3.1 W46R. (**A**) Correlations in the W46R mutant. The shortest distance between the residues L124 belonging to the chains A and C defining the gating at this level is denoted g124ac, and similarly, for Y132, it is designated g132ac. The upward movement of the slide helices of the chains A, B, C and D are denoted upa, upb, upc, upd, respectively. The tilt angle of the axes of the inner and outer helices from the perpendicular line to the membrane plane are denoted tiltin and tilton, respectively, where n is the name of the chain. Blue and red circles correspond to positive and negative correlations, respectively. The diameter and the color intensity of a circle are proportional to the strength of the correlation. (**B**) Definition of the inner and outer tilts (brown and blue, respectively). The upward movement of the slide helices is defined as in Figure 5B Constrictions points L124 and Y132 are shown. (**C**) Correlations in the case of KirBac3.1 WT.

More precisely, (i) the shortest distance *d46a36d, d46b36a,* and *d46c36b* (see Figure 6’s caption for definitions) are correlated with the upward movement of the chain A (*upa*); (ii) distances *d46c36b,* and *d46d36c* are correlated with the upward movement of the chain B (*upb*); (iii) the distances *d46d36c,* and *d46a36b,* are correlated with the upward movement of the chain C (*upc*). Altogether, this evidence points towards a clear correlation in the mutated protein between the upward movement of the slide helices and shortened distances between R46 and D36 residues belonging to the adjacent chain (n + 1 or n − 1). In the WT protein, which is mostly in flipped-in conformation, (98.68% of the relaxed MDeNM structures), such correlations are still visible but involve the adjacent chain (n + 1) (Figure 5C).

- Movement of the cytoplasmic domain

We analyze here the movement of the cytoplasmic domain since it has been considered an essential factor in gating [18,26,27,28,29]. To investigate its relation with the upward movement of the slide helices, the shortest distance between D35, the first residue of the slide helix, and R167, located at the top of the cytoplasmic domain on the CD-loop, was taken as a measure of the proximity of these two structural regions. Also, inter-chain distances were taken into account to corroborate the findings. Significant correlations were observed between these distances and the upward movement of the slide helices, as shown in Figure 5A. Furthermore, strong negative correlations are present between inter-chain distances and the upward movement of the slide helices to the membrane. When these distances decrease, the slide helices come closer to the membrane. More precisely, *d35a167b* and *d35d167a* are inversely correlated with the upward movement of the chain A (upa), and *d35b167c* and *d35c167d* are inversely correlated with a similar upward movement of the chain C (upc); similarly, *d35d167a* has an inverse correlation with the upward movement of chain D (upd). Interestingly, the WT protein structure displays the same type of correlations (Figure 5C).

For each monomer of the protein, the comparison of correlations involving the distance between residue 46 and residue 36 (*d46n36*n − 1) on the neighboring chain (n − 1) and the distance between residue 35 and residue 167 (*d35n167*n + 1) on the neighboring monomer (n + 1) for KirBac3.1 WT and KirBac W46R reveals clear differences. Indeed, these distances are correlated in the case of KirBac3.1 W46R but not in the case of the KirBac3.1 WT. We hypothesize that this is the consequence of a new interaction present in the W46R mutant but not in the WT protein between R46 and D36 on the slide helix. While in the WT, the residue D35 interacts with R167 on the same polypeptide chain, the mutation at position 46 creates an inter-chain interaction between D36 and R46. The slide helix is, therefore, held in place by two interactions in different directions. In the case of the WT, the interaction D35-R167 promotes the orientation of the slide helix towards the cytoplasmic domain, significantly reducing its upward movement. Conversely, the formation of the new R46-D36 interaction in the W46R favors the movement of the slide helices toward the transmembrane domain.

- Movement of the transmembrane helices

The correlation between the upward movement of the slide helix and the tilting of the inner and outer transmembrane helices may affect the channel’s gating at the constriction points L124 and Y132. Definition of the inner and outer tilts is shown in Figure 6B in red and blue, respectively, and Materials and Methods. The MDeNM sets for the mutant protein were analyzed to highlight correlations between structural determinants involved in the slide-helix movement and the tilting of the transmembrane helices (Figure 6A). High correlation values indicate the interplay between the inner and outer helices’ tilt angles with the movement of the slide helices. Similar correlations were obtained for the WT (Figure 6C).

In the center of the channel, two constriction points corresponding to residues L124 and Y132 are important for the gating as they control the passage of the potassium ions through the channel. In more detail, the gating at L124, represented by inter-chain distances *g124ac* and *g124bd* is correlated with the upward movement of the slide helices (upa,b,c,d). In addition, the *g124ac* distance is correlated with the tilt of the transmembrane helices (tiltin, tilton). Conversely, the opening of the gate at the level of the residue Y132 represented by the inter-chain distances *g132ac* and *g132bd* has an inverse correlation with the upward movement of the slide helices (upa,b,c,d) and the tilt of the transmembrane helices (tiltin, tilton). In the case of KirBac3.1 WT, data from the MDeNM set show no clear correlation between the gating at the level of residue L124, while the opening of the gate at the level of the residue Y132, specifically for chains B and D, is mainly correlated with the upward movement of the slide helices and the tilting of the transmembrane helices (conversely to KirBac3.1 W46R).

The upward motion of the slide helix causes the tilt of both transmembrane helices in such a way that the central portions of the transmembrane helices move away from the center of the channel (L124) while their cytoplasmic ends get closer to the bottom of the channel (Y132). This causes the increase of the opening of the channel at the level of L124 but a decrease in the channel’s opening at the level of Y132.

#### 2.3.4. Population of Open and Closed States Corresponding to the MDeNM Relaxed Structures

Note that in this work, a “closed” state is defined by a conduction pathway that is sterically occluded and an “open” state in which the pathway is sufficiently wide to accommodate at least a non-hydrated potassium ion. Four channel-gating states can be defined based on the open or closed conformation of the two main constriction points (L124 and Y132) as observed in the relaxed structures (for more details, see [18]): (1) Fully open state when the shortest distances at the two constriction points are larger than the ionic diameter of K^+^ (diameter of K^+^ ion considered 3.53 Å); (2) Fully closed state when both distances are smaller than the ionic diameter of K^+^; (3) Half-open state 1 when the gate at residue L124 is open and the gate at residue Y132 is closed; (4) Half-open state 2 when the gate at residue Y132 is open and the gate at residue L124 is closed. Interestingly, the fully open state structures constitute about 7.4% of the whole population for the mutant protein, a slightly higher percentage (0.5%) than KirBac3.1 WT (6.8% Table 2). Furthermore, we can notice that the gate at residue Y132, closer to the cytoplasmic domain, is more often closed than the gate at residue L124 (32.5% versus 10.8%, respectively). It follows that the residue Y132 in both protein WT and the W46R mutant protein is the most restrictive point of the ion pathway. A more significant observation is that the “gate” at L124 is more open in the case of the mutant W46R compared with WT (33% and 29%, respectively). Conversely, the gate at Y132 is less open in the mutant W46R than WT (11% and 14%, respectively).

**Figure 7:**
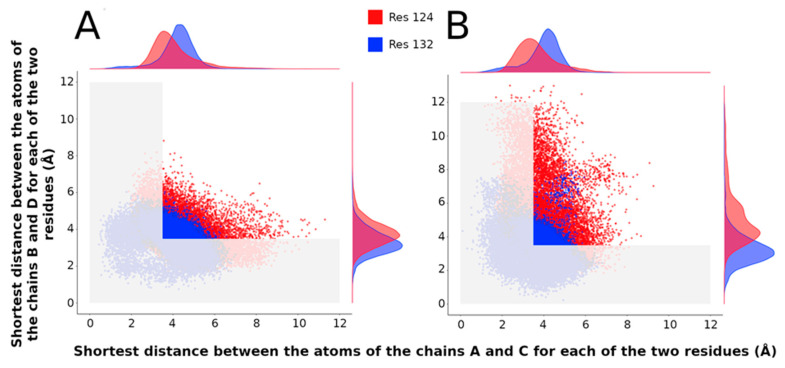
Gating at the two constriction points L124 and Y132. (**A**,**B**) Scatter plots of distances between Cα atoms of the residues 124 (in red) and 132 (in blue) for chains AC and BD. (**A**): For KirBac3.1 WT (**B**): For KirBac3.1 W46R.

More details about the amplitude of the channel opening at the gating points on the scatter plots of shortest distances between the opposite chains A and C and those between chains B and D at both constriction points over all the MDeNM relaxed structures are shown in Figure 7A,B for the WT and the W46R mutant proteins, respectively. The shortest distance between opposite chains at the level of the two constriction points is always lower than 15 Å for WT protein (Figure 7A) but higher than 15 Å for a small population of the simulated structures of the W46R mutant (Figure 7B). Interestingly, for this population, the opening is more prominent at residue Y132 compared with residue L124.

### 2.4. Functional Studies of the W46R Protein

**Figure 8:**
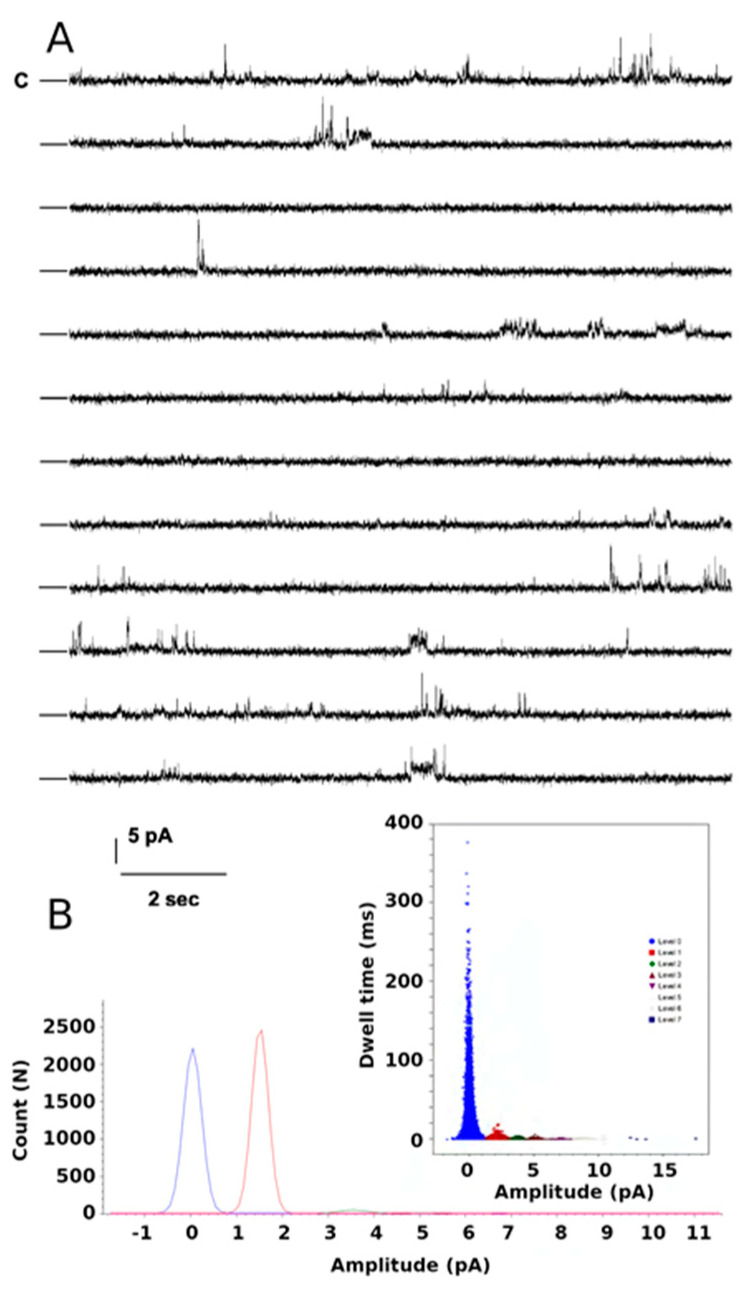
Single-channel recordings of the Kirbac W46R channels. (**A**) Current traces from 3 min consecutive recordings at +100 mV test potential. The closed state is labeled as C. (**B**) Amplitude histogram fits of all events for the selected seven levels gating between closed and open states during 3 min. Inset shows current amplitude values versus dwell time for all seven levels.

Purified W46R was incorporated in a preformed lipid bilayer. Figure 8A shows single-channel traces for a 3 min consecutive recording at +100 mV and channels gating with multiple substates as described for the Kirbac3.1 WT channels [18]. Analysis of the amplitude value versus the dwell confirms seven levels during this recording time (Figure 8B inset). Fits of the histogram of all levels versus current amplitude reveal a single-channel conductance value of 36 pS, similar to that recorded for the WT channels (47 pS at −80 mV [18]). From this recording, the open probability (Po) was 0.071 (Table 2). We performed recordings at other potentials that led to Po in the same order of magnitude (data not shown).

## 3. Discussion

### 3.1. Characteristics of Mammalian Kir6.2 Channel

Tryptophan at residue 68 of the Kir6.2 subunit is critical for correct channel function [13]. Most of the W68 mutants in Kir6.2 cause a gain in channel function. In all Kir crystal structures, the residue corresponding to W68 in Kir6.2 lies at the N-terminal end of TM1, close to the hinge region that leads to the slide helix. As seen in most of the X-ray structures of WT Kir channels, this highly conserved residue is in the flipped-in conformation, tightly packed against the C-terminal end of TM2 in a hydrophobic cluster (Figure S3 and Video S1), with the tryptophan side chain pointing toward the center of the channel. The residue also interacts with I131, F135, and R134 at the bottom of the TM2. These interactions favor the closed state of the channel. However, it has been suggested that the flipped-out conformation of W68 is required for the movement of TM2 that leads to an open state [13].

Multiple residues in Kir6.2 (more than 70) are known to cause neonatal diabetes mellitus. They can be divided into three classes: the class of residues that act directly by increasing the opening probability of the channel, the class of residues that participate in ATP binding (including those who lie in close vicinity), and the residues that are acting as a conformational relay by which ATP binding is translated into pore closure [6].

### 3.2. KirBac3.1 W46R: A Good Model for Studying Analogous W68R Mutation in Mammalian Kir6.2

Here we investigate the effect of one particular mutation on the bacterial homolog KirBac3.1 channel and compare our findings to the mammalian Kir6.2. We support the idea that the KirBac3.1 W46R mutant we characterized here may represent a good model for studying the analogous mutation in the human channel and that it can help provide insights into the molecular mechanisms responsible for the gain of function of the channel. Therefore, we solved the structure of KirBac3.1 W46R at 2.8 Å resolution and used this model to investigate the dynamics of the protein. Structural comparison between Kir6.2 and KirBac3.1 is shown in Figure S5.

In the case of the mutant KirBac3.1 W46R, replacing the hydrophobic residue tryptophan with a positively charged arginine in this region is likely to have a destabilizing effect. Analysis of the X-ray structure of KirBac3.1 W46R and the modeled structure from 2WLJ, with an rmsd of 1.712 Å between the two structures, shows that the residue R46 does not point toward the center of the channel but resembles the flipped-out conformation of W46 pointing away from the channel pore. As a consequence, the previously described interaction with the cytoplasmic end of the TM2 helices and the hydrophobic cluster around W46 are disrupted (this hydrophobic cluster is also present in Kir6.1 and Kir6.2 [30]), and the R46 residue forms a new network of interactions with the slide helix of the neighboring subunit (n – 1) involving D36 and W38 residues. Note that D36 is also highly conserved among the Kir channels (Figure 1C).

W46R does not appear to increase the opening probability of the KirBac3.1 channel.

Our functional and structural experiments showed no significant difference between the opening probabilities of the WT and mutated channels. The fully open state structures constitute about 7.4% of the whole population, compared to KirBac3.1 WT (6.8%, Table 2). The functional studies confirm these data and show that the open probability (Po) for the W46R is even slightly smaller than that of the WT (7.1% compared with 9.9%, respectively). The HDX studies show that the only region that is less flexible compared with the WT structure is at the cytoplasmic bottom end of the TM1, including the R46 mutation, suggesting that the network of interactions formed between the mutated residue R46 and the slide helix increases the rigidity of this protein segment. This suggests that the mutation does not affect the channel’s intrinsic open probability, as other neonatal diabetes mutations do with the human isoform [6].

Gating transitions of KirBac3.1 W46R revealed by molecular dynamics simulations.

Despite the similar open probabilities between the KirBac3.1 WT and W46R, the mutant describes a different behavior. Indeed, the detailed dynamical view of the entire mutant channel during the gating associated with the open/closed transition was uncovered by *in silico* studies that show a sequence of molecular events that, due to lack of interactions between TM1 and TM2, trigger the opening of the channel. In the WT state, the tryptophan residue at position 46 forms contacts with transmembrane domain 2 (TM2) that hamper its movement and gate opening. In the W46R mutant, the arginine side chain is flipped away from the channel pore, enabling the movement of TM2 and the establishment of new contacts with the slide helix of the neighboring subunit (n − 1) that aid in the gating mechanism and increased open state stability. The gating mechanism can be summarized as a succession of events: (i) R46 interacts with slide helix (D36); (ii) The upward movement of the slide helices induces the bending of the TM1 and TM2; (iii) this, in turn, causes a larger opening of the channel at the level of the residue L124 but a closer interaction between the subunits at the level of residue Y132. Interestingly, some of these events can be correlated between the different chains, unlike what happens for the WT [18] where there is no correlation between the different chains. Therefore, these particular synchronized steps could help for the opening of the channel.

To further assess the flexibility of the structures of KirBac3.1 WT and the W46R mutant, we compared the rms fluctuations between the two structures. The results are shown in Figure S4 where KirBac3.1 WT seems, in general, more flexible than the mutant KirBac3.1 W46R. However, the stretch that includes the very end of the slide helix, R46 mutation, and the bottom half of the TM1 external helix (aa 43–56) is not as flexible as the WT, as noted in HDX-MS.

### 3.3. Suggestions to Kir6.2 by Analogy

W68 does not directly participate in ATP binding. The ATP-binding site on Kir6.2 lies at the interface between two Kir6.2 subunits and close to the L0 loop of SUR. While the resolution of the cryo-electron microscopy density does not allow precise determination of atomic-level ATP–protein interactions [31,32] (see structures PDB 6C3P and PDB 6C03), when combined with the finding of functional studies, it provides an accurate model of the ATP-binding site. The amino acids belonging to the ATP-binding site using Kir6.2 numbering are R50 and N48 for chain A, and I182, K185, Y330, F333, and G334 for chain B. The other residues lying nearby (such as H46, E51, Q52, R201, and G53) are important for stabilizing the interaction with ATP. Even if residue 68 was previously reported as not directly implicated in the interaction with ATP, the introduction of a mutation could lead to a destabilization of the nearby region responsible for ATP binding, as reported for other neonatal diabetes mutations [6]. This destabilization of the closed channel by disrupting the nearby hydrophobic cluster in the closed state (Figure S3) has been reported previously for other mutations such as K170 (K170T) and I167 (I167L) in Kir6.2. These mutations result in neonatal diabetes by reducing inhibition by ATP [33,34]. Various mutations at residue T171 can also cause a significant increase in the open probability [35]. All these residues are known to interact with W68.

Therefore, it is possible that the substitution of W68 by R may result in a pathogenic reduction of ATP sensitivity (inhibition) by disrupting the normally present interaction between the bottom of TM1 and the slide helix. This is in line with the reduced sensitivity toward ATP inhibition already reported for the corresponding human mutant W68R, which also has been characterized by increased currents [13]. Furthermore, W68 could be necessary for the conformational relay by which ATP binding in mammalian Kir6.2 is translated into pore closure. The mutation could then alleviate the functional inhibition due to ATP binding. Although our results on the KirBac3.1 mutant channel do not allow us to conclude on this aspect, they provide structural insights that may be useful for understanding Kir6.2-associated disorders.

In conclusion, W68 represents a highly conserved residue among the Kir channels, also conserved in the human K-ATP channels. Here we show, by analogy with the KirBac3.1 W46R mutant, that W68 and its network of interactions are crucial for the activity of this channel, as it is involved in controlling channel gating. In the human channel, mutations of this residue lead to neonatal diabetes. Our findings may represent the basis for further investigations on the homologous human K-ATP channel to provide novel insights into the molecular mechanisms responsible for the pathological gain of function of the channel.

## 4. Materials and Methods

### 4.1. Experimental Data

#### 4.1.1. Protein Expression and Purification

The W46R mutation was introduced using site-directed mutagenesis into a synthetic KirBac3.1 gene inserted in pET30a. The open reading frame of this construct had been codon-optimized for expression in *Escherichia coli*. Protein expression and purification of this mutant channel were performed as outlined before [36]. Briefly, after cell disruption by the French press, the protein was directly solubilized with 45 mM DM (Decyl β-D-maltopyranoside), centrifuged, and the supernatant was loaded onto a Co^2+^ affinity column. The protein was promptly purified on a Superdex 200 10/300 column pre-equilibrated with 2 mM TriDM buffer. Concentrated preparations (1–2.5 mg/mL) of purified proteins were stored at −80 °C in buffer containing 20 mM Tris, pH 7.4, 150 mM KCl, and 0.2 mM TriDM.

#### 4.1.2. Protein Crystallization

We used the experimental procedures and equipment available in the crystallography core facility at the Institut Pasteur, Paris, [37] to perform the crystallization screening trials. Briefly, with a Mosquito™ nanoliter-dispensing system (TTP Labtech), we set up crystallization sitting drops of 400 nL containing a 1:1 mixture of protein and crystallization solutions (672 different commercially available conditions). The protein drop was equilibrated against a 150 μL reservoir in multiwell plates (Greiner Bio-One). The crystallization plates were stored at 18 °C in a RockImager (Formulatrix) automated imaging system to monitor crystal growth. Manual optimization was performed in Linbro plates with the hanging-drop method by mixing 2 µL of KirBac3.1 W46R protein at 10 mg/mL with 2 µL of reservoir solution. The best crystals were obtained under the conditions containing 15% (*w*/*v*) PEG- MME 5k, 15% (*v/v*) glycerol, 1 M LiCl, and 100 mM MES pH 6.5. For data collection, the crystals were flash-cooled in liquid nitrogen using the reservoir solution as cryo-protectant.

- Data collection and structure determination

The X-ray diffraction data were collected at 100K on the PROXIMA-1 beamline (Synchrotron SOLEIL, St Aubin, France) using a Dectris pixel-array PILATUS 6M detector at a wavelength of 1.07114 Å to a resolution of 2.80 Å. The diffraction images were integrated with the program XDS [38]. The KirBac3.1 W46R crystals belong to the P2_1_2_1_2 space group with cell dimensions of 106.77 Å 113.98 Å 89.18 Å. Molecular replacement was carried out with Phaser (CCP4 suite), using a search model derived from the closed structure of KirBac3.1 closed state at 2.85 Å (PDB 2WLJ). Five percent of the reflections were set aside in the free R set. The asymmetric unit has a solvent content of 69.4% and contains two polypeptide molecules. The model was refined in real space interactively using Coot [39] and refined using BUSTER-TNT [40], which included a final round of translation, vibration, and screw-rotation (TLS) anisotropic refinement, as implemented in BUSTER-TNT. An additional restrained refinement was carried out using REFMACS [41]. The final model contains two polypeptide chains with 276 and 265 residues out of 301, respectively. The model also contains 23 solvent molecules and four ions (three K^+^ and one Mg^2+^). The final model having 99.3% of all residues in the favored or allowed regions of the Ramachandran plot was validated using MolProbity [42]. The crystals’ parameters, data statistics, and refinement parameters are shown in Table 1. All structural figures were generated with PyMOL (version 1.3r1). The PDB accession code of this structure is 7ADI.

#### 4.1.3. Pepsin Digestion, Hydrogen/Deuterium Exchange Approach Coupled to Mass Spectrometry (HDX-MS) and HPLC

Peptide separation of this mutant channel was performed as outlined before [18]. Briefly, all protein digestions in solution were performed in an ice bath at 0 °C. Protease solutions were prepared in 500 mM glycine (pH 2.2). KirBac3.1 protein samples were digested in the same buffer for 2–5 min using a protease/substrate ratio of 1:1 or 1:10 (wt/wt) for pepsin and nepenthesin, respectively, either in solution or immobilized on a resin. The increase in digestion time did not affect the proteolysis.

HDX-MS reactions were carried out on KirBac3.1 W46R at a protein concentration of about 10 μM. The reaction was initiated by a 10× dilution of the protein samples (10 μL) into a deuterated buffer containing 50 mM KCl and 0.2 mM TriDM. The time course of the HDX was followed over a 20 min period by sequential withdrawing 120 μL of deuterated samples, which were immediately added to 26 μL of quenching buffer (8 M guanidium chloride, 500 mM glycine HCl, pH 2.2), rapidly mixed, and flash-frozen in liquid nitrogen. After protease digestion in solution or on column in an ice bath at 0 °C, the peptides were loaded onto a peptideMicroTrap (Michrom Bioresources) column and washed with 0.03% TFA in water (HPLC solution A). They were then separated on a reversed-phase C12 column as previously described [20] during 26 min. The column was connected to the electrospray source of the mass spectrometers until 30% (vol/vol). The valves, trap cartridge, and column were cooled to 0 °C by immersion in an ice bath as outlined before [20]. The tandem MS (mapping) analyses were performed on an ion trap mass spectrometer (Esquire 3000+; Bruker Daltonics) to identify the peptides after separation on HPLC. Mass measurements and local deuteration kinetics analysis were performed on a time-of-flight (TOF) mass spectrometer (6210; Agilent Technologies) equipped with an electrospray source. Each deuteration experiment was performed in triplicate. Data were processed with MassHunter software (Agilent Technologies). The qualitative analysis, the deconvolution, and calculation of the average masses were carried out in Magtran [43].

#### 4.1.4. Functional Studies of the KirBac W46R Protein in Black Lipid Membrane

Purified KirBac3.1 W46R (130 µg/mL) in DM (decylmaltoside) detergent (1.5 mM) was added to the upper chamber (150 µL) to a preformed bilayer. The Orbit mini apparatus was used (Nanion, Germany, horizontal planar lipid bilayer system), with two chambers separated by a partition with a 100-µm hole where the lipid bilayer is formed by 1,2-diphytanoyl-sn-glycero-3-phosphocholine (DPhPC,10–30 pF). The lower chamber contained 150 mM KCl, 10 mM MOPS, pH 7.4. After membrane bilayer formation, the upper chamber solution was changed to 150 mM KCl, 10 mM MOPS pH 8. Currents were recorded using Elements Data Reader (Nanion, Germany) and analyzed using Clampfit (Axon Instrument Inc. Molecular devices, CA, USA) software, sampled at 100 µs, and filtered at 1.25 kHz. The recording was performed at 24 °C using a Nanion temperature control unit.

### 4.2. Theoretical Data

#### 4.2.1. Modeling and Dynamics

The X-ray structure of KirBac3.1 with an atomic resolution of 2.6 Å (PDB 2WLJ) [14] was used to model the complete WT structure. As the first 34 residues were missing in the X-ray structure, this segment was excluded from our model, and the missing atoms were constructed using the CHARMM program [44]. Residues 1 to 34 of the protein are not likely to be involved in the gating process. The tetrameric complex was built by applying a twofold symmetry axis for this structure using the Protein Interface Surface and Assemblies software through Pisa [45] at EBI. The W46R mutation was achieved with Chimera [46], considering the modeled WT structure. The ionization states of residues in both structures were established using PROPKA3 [47,48]. A complete system comprising a DOPC membrane, TIP3P type water molecules [49], and KCl ions at 150 mM concentration within a box of 116.243 Å × 116.243 Å × 133.255 Å was built using the membrane-builder module of Charmm-Gui webserver [50,51]. This web server uses the Orientations of Proteins in Membranes (OPM) [52] software to imbed the protein into the membrane. We used the charmm36 force field [53]. The WT and mutant structures were first energy minimized, then further relaxed by carrying out short molecular dynamics simulations for 600 ps at a constant temperature of 300 K using the Nose-Hoover thermostat and constant pressure of 1 atm using the Langevin piston. Such a structure constituted the starting point for the normal mode calculations and MDeNM simulations. The Leapfrog Verlet algorithm was used for propagating the movement. The cut-on and cut-off distances for nonbonded interactions were set at 10 Å and 12 Å, respectively, and between these values, a switching function was applied [18].

#### 4.2.2. Normal Modes

The first 200 lowest frequency normal modes (NMs) of the structure KirBac3.1 W46R structure were calculated with the DIMB method [54] in CHARMM. A switching function was applied to the nonbonded interactions by setting a cut-on distance of 10 Å and a cut-off of 12 Å. A distance-dependent dielectric constant of 2r (r being the interatomic distance) was used. The minimization was carried out under harmonic restraints applied to atomic positions, which were gradually reduced and finally continued without restraints by performing 50,000 steps of CG (conjugate gradient) followed by ABNR (Adopted Basis Newton–Raphson) minimization until reaching an RMS energy gradient of 10^–5^ Kcal mol^−1^ Å^−1^. The NMs of this minimized structure were then computed. We selected from these modes those directly involved in the channel gating to be used in MDeNM (molecular dynamics using excited normal modes) simulations, following the same procedure as for KirBac3.1 WT [18]. Eight contributing modes were selected, with the ranking numbers 85, 86 106, 150, 153, 154, 166, and 171 ordered in increasing frequencies. We selected these modes according to their contributions to the opening/closing of the channel by following a list of criteria as explained in [18,23].

#### 4.2.3. Molecular Dynamics Using Excited Normal Modes

The MDeNM method [22] was applied to explore the large-scale conformational changes in the protein. This method allows an extensive conformational exploration in the normal mode space coupling global and local motions. It consists of propagating the movements by kinetic excitations along the different linear combinations of mode vectors in MD simulations, each one of these simulations constituting a replica. These combined vectors are chosen to point to different regions of the normal mode space allowing an extensive exploration. Substantial conformational changes could then be explored in a series of relatively short MD simulations to obtain a set of structures that would require at least simulations in the range of ms with conventional MD. Our study considered 130 replicas for KirBac3.1 W46R, which allowed for an extensive conformational sampling (total simulation time = 0.4 ns × 130 replicas = 52 ns). A filtering procedure was used to determine the mode combinations allowing an isotropic distribution of the vectors [18]. We considered for each replica a series of 7 kinetic energy excitations, each lasting 1.5 ps, and corresponding to an 8 K temperature increase of the protein. These simulations were carried out with the CHARMM program using a dedicated script (available upon request).

- Relaxation of the excited MDeNM structures

MDeNM provided 910 excited structures (7 excitations for every 130 replicas). This set of structures contained a few similar structures despite the rms filtering. Therefore, we proceeded to a clustering of the structures based on the channel rmsd (1.0 Å for KirBac3.1 W46R), carried out with the clustering tool VMD, yielding 85 clusters. The structure closest to the average structure within each cluster was chosen as the representative of the group. They were then relaxed during 0.4 ns standard molecular dynamics performed with NAMD using the same force field as before. Finally, we considered the last 0.3 ns of the trajectories in which the remaining excitation energy is substantially dissipated, providing 25,415 structures used in our analysis.

#### 4.2.4. Interaction Networks

The interaction networks around the residue 46 in KirBac3.1 WT (flipped-in and flipped-out configurations) and KirBac3.1 W46R mutant were determined using the Ring Web server of the University of Padova [55] and Cytoscape [56]. The nodes of the network correspond to the residues, and the edges to the physicochemical interactions, hydrogen bonds, or Van der Waals interactions. In addition, specific sub-networks were established by Cytoscape, which reads the files created by Ring and extracts information related to the residues of interest and their neighbors and the interactions between them.

#### 4.2.5. Structural Determinants

Accessible surface area (ASA) was calculated with a Tcl script in VMD [57], the shortest distance between residues with the CHARMM program [44], and the Pearson correlation coefficients with the cor module of the R software (https://www.R-project.org/, accessed on 1 March 2021).

- Upward angle of the slide helices

The upward angle of a given chain’s slide helix (residues from T43 to D35) is defined as the angle between its axis and the membrane plane (Figure 5B, in pink). It measures its proximity to the membrane; the higher the value, the closer to the membrane. The upward angles of the A, B, C, and D chains’ slide helices are denoted upa, upb, upc, and upd, respectively.

- Tilt angle of the transmembrane helices

The tilt angle of a transmembrane helix is defined as the angle between its axis and the perpendicular to the membrane plane (Z-axis) (Figure 6B). The inner helix of each chain is delimited from A109 to F135 and the outer helix from the residue 46 (in W/R46 or WT) to Cys71. The tilt angles of the inner transmembrane (TM) helices are named inclia, inclib, inclic, inclid for the chains A, B, C, and D, respectively, and similarly incloa, inclob, incloc, and inclod concerning the outer TM helices.

## Figures and Tables

**Table 1:** Data collection and structure refinement statistics for KirBac W46R.

**Data Collection**	
Space group	P2_1_2_1_2
Cell dimensions (Å)	106.77, 113.98, 89.18
Cell angles	90° 90° 90°
Resolution (Å)	48.34–2.80
Completeness (%)	99.6
I/σ (I)	19.67 (1.31 in the high-resolution shell)
Clashscore	8
Ramachandran outliers	1.7%
Side-chain outliers	9.3%
**Refinement with REFMAC 5.8.0258**	
R, R free	0.222, 0.287
R free test set	1372 reflections (5%)
Wilson B-factors (Å^2^)	89.3
Total number of atoms	4341
Average B-factors, all atoms (Å^2^)	97.464
RMS of Z score of bond lengths	0.70
RMS of Z score of bond angles	0.89

**Table 2:** Populations (in percentages) of different opening states in the relaxed structures of KirBac W46R obtained through MDeNM simulations and single-channel recording of the mutant and KirBac3.1WT.

Channel States	Kirbac3.1 WT (%) (16)	KirBac3.1 W46R (%)
Fully open	6.8	7.4
Fully closed	50.2	49.3
Half open 1 (124 open, 132 closed)	28.8	32.5
Half open 2 (132 open, 124 closed)	14.2	10.8
Current recordings of KirBac3.1 in planar lipid bilayers	9.9 (±1.3, *n* = 1803)	7.1

## Data Availability

Accession codes. The atomic coordinates of the KirBac3.1 W46R structure have been deposited in the Protein Data Bank under the accession code 7ADI.

## Supplementary Materials

Supplementary Materials are available online at https://www.mdpi.com/article/10.3390/ijms23010335/s1.
