# Supplementary material for: Integrative Study of the Structural and Dynamical Properties of a KirBac3.1 Mutant: Functional Implication of a Highly Conserved Tryptophan in the Transmembrane Domain
Source: Int J Mol Sci. 2021 Dec 29;23(1):335. doi: 10.3390/ijms23010335 (PMC8745282; doi:10.3390/ijms23010335)
Supplement: Supplementary file 1 [file ijms-23-00335-s001.zip › ijms-1490025-supplementary/ijms-1490025-supplementary.pdf]

Supplementary

Integrative study of the structural and dynamical properties of a KirBac3.1 mutant: functional implication of a highly conserved tryptophan in the transmembrane domain.

Charline Fagnen[1,2], *Ludovic Bannwarth*[1], *Iman Oubella*[1], *Dania Zuniga*[1], *Ahmed Haouz*[3], *Eric Forest*[4], *Rosa Scala*[5], *Saïd Bendahhou*[5], *Rita De Zorzi*[6], *David Perahia*[2] *and Catherine Vénien-Bryan*[1]*

[1] IMPMC, UMR 7590, CNRS, Muséum National d'Histoire Naturelle, Sorbonne Université, 75005 Paris, France
[2] Laboratoire de Biologie et Pharmacologie Appliquée, Ecole Normale Supérieure Paris-Saclay, 4 Ave. des Sciences, 91190 Gif-sur-Yvette, France
[3] Institut Pasteur, C2RT-Plate-forme de Cristallographie, CNRS-UMR3528, 75724 Paris, France
[4] IBS University Grenoble Alpes, CEA, CNRS 38044 Grenoble, France
[5] University Côte d'Azur, CNRS UMR7370, LP2M, Labex ICST, Faculté de Médecine, Nice, France.
[6] Department of Chemical and Pharmaceutical Sciences, University of Trieste, Via Licio Giorgeri 1, 34127, Trieste, Italy

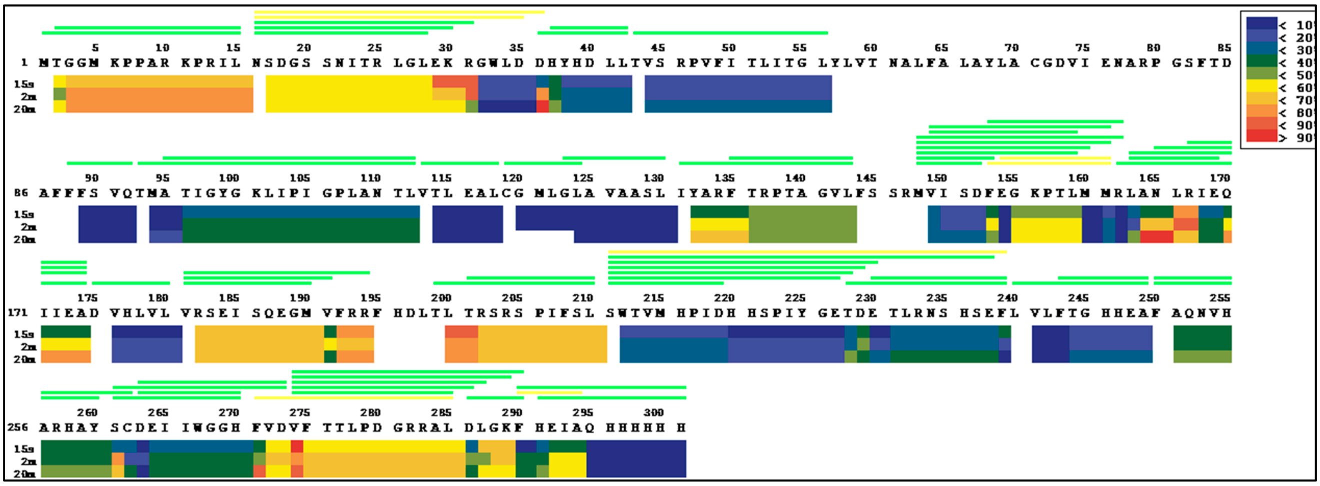

Figure S1 Heat Map of the KirBac3.1 W46R mutant. Deuteration maps of KirBac3.1 peptides at different times up to 20 minutes deuteration. The color key indicates the HDX level.

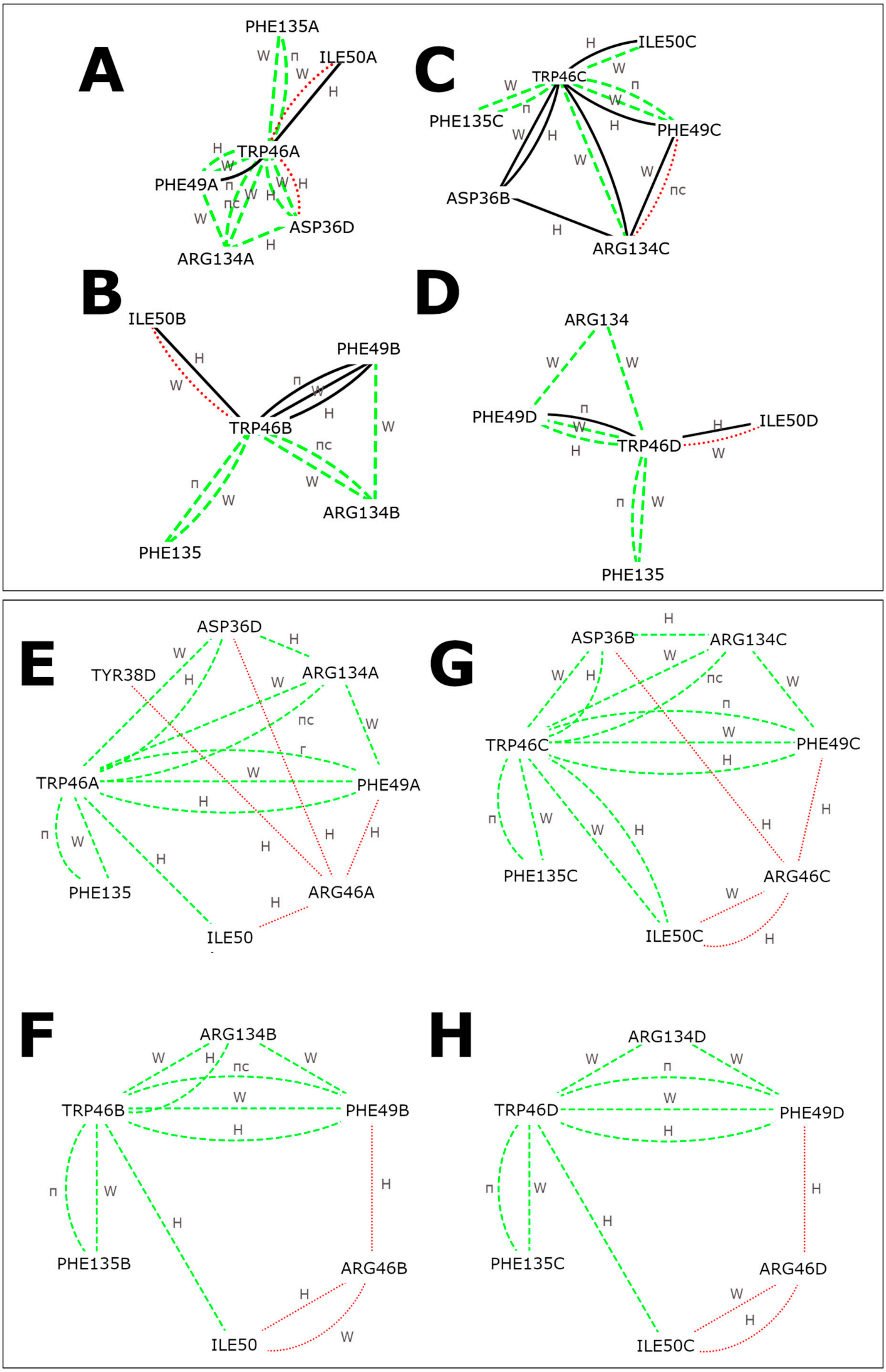
A
PHE135A
ILE50A
TRP46A
PHE49A
ASP36D
ARG134A
C
ILE50C
TRP46C
PHE135C
PHE49C
ASP36B
ARG134C
B
ILE50B
PHE49B
TRP46B
ARG134B
PHE135
D
ARG134
PHE49D
TRP46D
ILE50D
PHE135
E
ASP36D
TYR38D
ARG134A
TRP46A
PHE49A
PHE135
ARG46A
ILE50
G
ASP36B
ARG134C
TRP46C
PHE49C
PHE135C
ARG46C
ILE50C
F
ARG134B
TRP46B
PHE49B
PHE135B
ARG46B
ILE50
H
ARG134D
TRP46D
PHE49D
PHE135C
ARG46D
ILE50C

Figure S2 Detailed overview of the network of interactions around Trp46 (KirBac3.1 WT) and Arg46 (KirBac3.1 W46R). A, B, C and D: same as Figure 3 D,E,F and G i.e. : KirBac3.1 WT: Comparison of the interaction network of residue Trp46 in flipped-in and flipped-out conformation for the chains A (panel A), B (panel B), C (panel S), D (panel D). The green dotted lines indicate the interactions present in the flipped-in configuration but absent in the flipped-out one and red dotted lines those present in the flipped-out configuration but absent in flipped-in one. Black lines are shared between the two conformers. **F, G, H and I:** same as Figure 3 H, I, J and Y i. e. **:** Comparison of the interaction network of residue Trp46 flipped-in (KirBac3.1 WT) and of residue Arg46 (KirBac3.1 W46R) for the chains A (panel H), B (panel I), C (panel J), D (panel K). The green dotted lines indicate the interactions present in KirBac3.1 WT but absent in W46R mutant, and the red dotted lines the interactions present in the W46R mutant but absent in KirBac3.1 WT. The symbols along the edges indicate the interaction type: H for hydrogen bond, W for Van der Waals interaction, π for π-π interaction, and πc for π-cation interaction

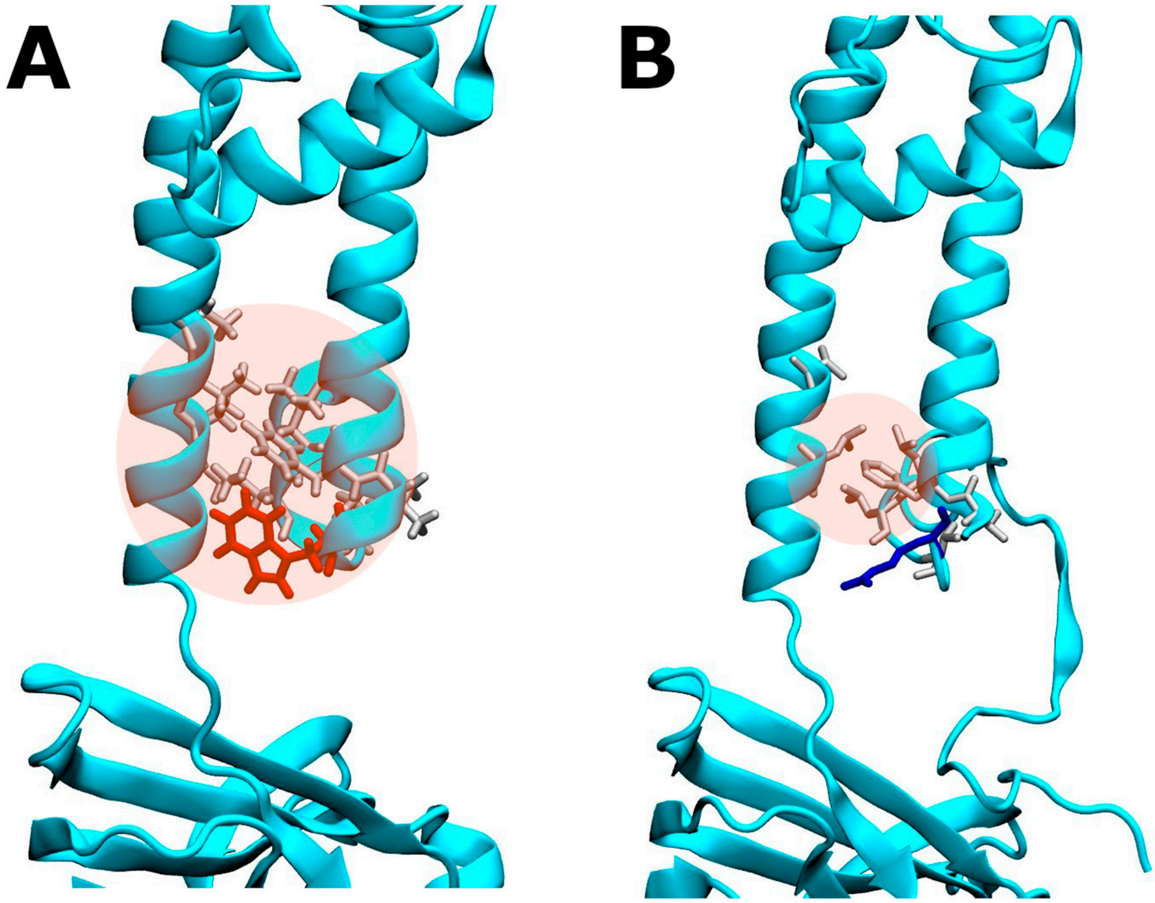

Figure S3 Hydrophobic cluster in KirBac3.1 WT made of residues in TM1 (Trp46, Val48, Phe49,), TM2 (Val126, Leu130,) and slide helix (Leu41, leu42, Val44) (A) and KirBac3.1W46R (B). W46 and R46 are in red and blue, respectively. Grey residues represented the hydrophobic residues close to the mutation, and the orange area circles the hydrophobic cluster of the two systems to illustrate them.

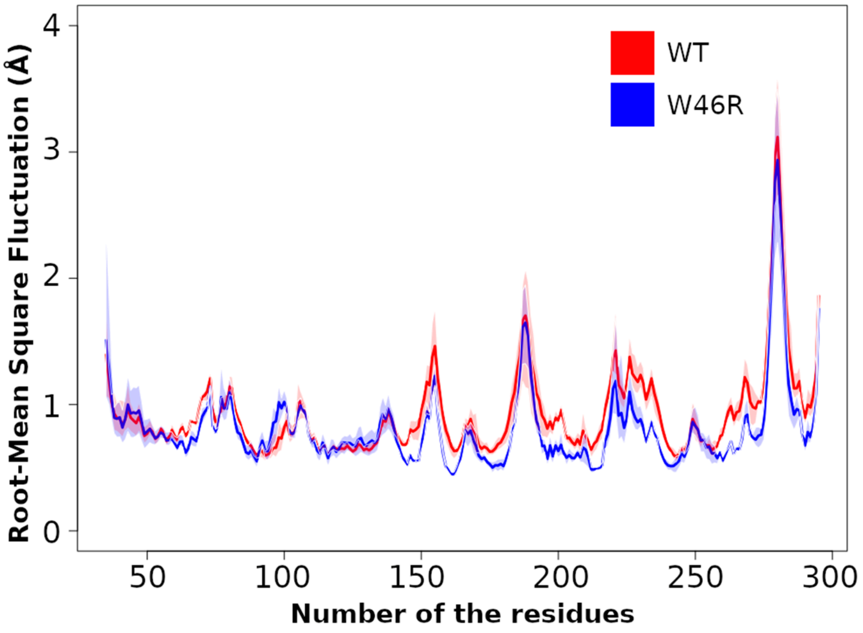

Figure S4: Root-Mean Square Fluctuation by residue computed on all relaxed structures from MDeNM in red and blue for KirBac3.1 WT and KirBac3.1 W46R, respectively.

.

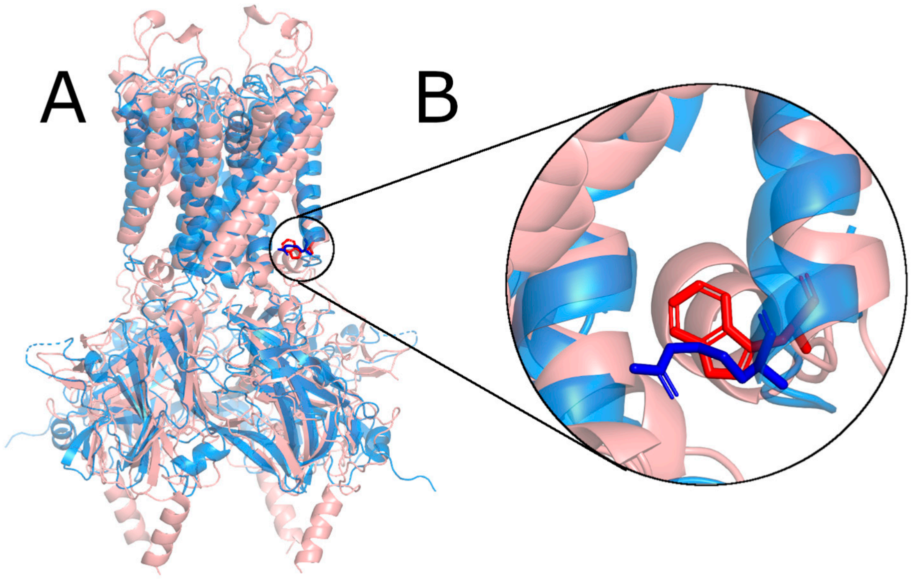

Figure S5: Structural comparison between the two structures Kir6.2 (red) and KirBac3.1W46R (blue); B: zoom on the mutation.
